# Neural oscillations associated with auditory duration maintenance in working memory

**DOI:** 10.1038/s41598-017-06078-2

**Published:** 2017-07-18

**Authors:** Xiaolin Yu, Youguo Chen, Junjie Qiu, Xiang Li, Xiting Huang

**Affiliations:** 1 Key Laboratory of Cognition and Personality (SWU), Ministry of Education, Chongqing, 400715 China; 2 Faculty of Psychology, Southwest University, Chongqing, 400715 China

## Abstract

The neural representation of auditory duration remains unknown. Here, we used electroencephalogram (EEG) recordings to investigate neural oscillations during the maintenance of auditory duration in working memory (WM). EEG analyses indicated that the auditory duration length was not associated with changes in the theta band amplitude, whereas the alpha band amplitudes during 3-s and 4-s auditory duration conditions were lower than during the 1-s and 2-s conditions. Moreover, the alpha band amplitude and accuracy were positively correlated in the 2-s duration condition. We also found that the neural representation of auditory duration is segmented, with a critical threshold point of approximately 2 s, which is shorter than that for visual duration (3 s). The results emphasised the involvement of the alpha band in auditory duration maintenance in WM. Our study’s findings indicate that different internal representations of auditory durations are maintained in WM below and above 2 s from the perspective of electrophysiology. Additionally, the critical threshold point is related to the sensory modality of duration.

## Introduction

Time is a basic abstract concept that humans use to precisely process temporal information. Everyone uses temporal information in his or her daily life without explicitly thinking about it. However, the nature of time itself may be best explained by Augustine, who said, ‘what then is time? If no one asks me, I know; if I wish to explain it to one that asketh, I know not’^1^. A critical element of temporal information processing is working memory (WM), which involves short-term storage and the online manipulation of information^2^. WM can maintain and manipulate many types of information, such as digits, letters, words, locations, images, etc.

Few studies have investigated the maintenance or manipulation of temporal information in WM^3^ or the neural representation of duration in WM for different sensory modalities^4–8^. Behavioural studies have revealed that two types of memory subsystems—visuospatial sketchpad and phonological loop—maintain visual duration and auditory duration, respectively^7, 9, 10^. However, several limitations prohibit behavioural studies from examining the internal representation of duration in WM. In contrast, neurophysiological studies have indicated that neural oscillation is critically involved in the neuronal dynamics required for sustaining WM representations^11–13^. Compared with other frequencies, theta and alpha bands are routinely delineated in neural oscillation studies of WM. Previous studies have reported correlations between WM load and theta oscillations (4–8 Hz) over prefrontal regions or alpha oscillation (8–12 Hz) over posterior sites^14–20^. Some studies have suggested that alpha oscillations are involved in inhibiting the cortical areas representing information that is no-longer-relevant^18, 21, 22^. In contrast, others have suggested that the alpha oscillation is associated with the successful maintenance of item information^3, 17, 23^. However, more research is needed to specifically investigate the neural representation of temporal WM.

Recently, using electroencephalogram (EEG) recordings to examine the neural oscillatory correlates of visual duration in WM, Chen and colleagues^3^ found that alpha activity rather than theta activity was involved in the maintenance of visual duration in WM. Additionally, different alpha activities occurred during WM maintenance below and above 3 s, which provides electrophysiological evidence for the perspective of segmented duration representation^24, 25^. They argued that the neural representations of different lengths of durations are distinct. Thus, an estimated critical threshold exists. Durations below the critical threshold will be recognised as the ‘subjective present’, and they typically should result in the perception of a coherent experience or temporal gestalt^25^. In contrast, durations above the threshold would not result in the perception of a single unit because of disintegration^24^. There is some evidence to support this claim, e.g. Elbert *et al*.^26^ employed event-related potentials (ERPs) and found that there was a critical threshold (3 s) for the accuracy of visual duration reproduction. Additionally, it was accompanied by a slow negative wave named contingent negative variation (CNV) during reproduction durations within the threshold, whereas the CNV was reduced or even absent when durations were beyond the threshold. Moreover, a functional magnetic resonance imaging showed that the motor system and default mode network respectively process durations below and above 2 s^27^. Further indications for the perspective of segmented duration representation are derived from the perception of rhythmic coherence (e.g. it becomes impossible about the perception of rhythm if the tones are separated by intervals exceeding 3 s in a regular sequence^28, 29^) and sensorimotor synchronization (e.g. the appropriate synchronization between a regular sequence of beats and corresponding motor acts breaks down when the interstimulus interval between the beats exceeds 2~3 s^30^).

However, the neural representation of auditory duration remains unknown, and thus is the focus of our study. It is an important characteristic of duration perception that subjectively perceived durations vary between different sensory modalities (e.g. auditory or visual). Many studies showed that auditory signals are often judged as longer than visual signals for a given duration^31–35^, for example, Wearden *et al*.^34^ used duration bisection task and verbal estimation found that auditory signals appeared longer than visual signals in all cases, and the effect was greater at longer stimulus durations. They suggested that auditory signals drive an internal clock at a faster rate than visual signals^32, 33^. Similar auditory/visual difference in duration judgment was also observed in 5- and 8-year-old children as well as young adults^35^. But this modality difference was more obvious for children. They believed that it is more automatic for the processing of auditory than visual signals for all age groups, however, visual signals require more attentional resources. Then it is more difficult for children to process visual signals than adults because of the limited attentional abilities. Therefore, considering that auditory signals are often judged to be longer than visual ones^31–35^, we hypothesised that the neural representation of auditory duration in WM is also segmented, but the critical threshold may be shorter than that for visual duration.

**Figure 1:**
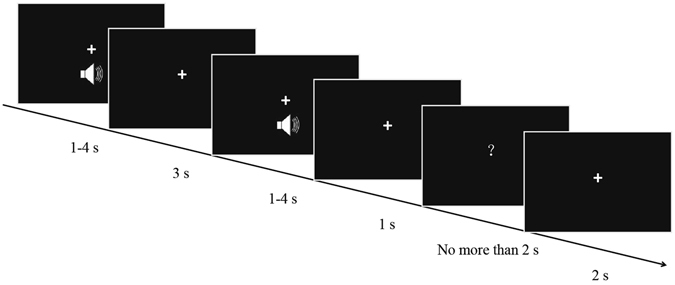
Trial sequences and durations of each screen presentation. Auditory stimuli were randomly presented for 1, 2, 3, or 4 s. Each trial starts with a pure tone (sample), followed by a 3-s interval (the delay/maintenance phase). Next, a second pure tone (probe) is presented. Participants press ‘1’, ‘2’, or ‘3’ correspondingly after estimating the duration of the second pure tone (probe) as shorter, equal to, or longer than the first tone (sample).

Here, we applied a matching-to-sample task (Fig. 1), following the study of Chen *et al*.^3^, to investigate the theta and alpha oscillation correlates of auditory duration maintenance in WM. The advantage of this paradigm includes the separation of the encoding, maintenance, and decision stages of temporal information^36, 37^. Subjects were required to maintain one duration (1 s, 2 s, 3 s, and 4 s) in WM. As for the theta band, we hypothesised that the auditory duration length would not be associated with changes in the theta band amplitude, because the theta band mainly reflects the maintenance of temporal order information^23, 38^. As mentioned earlier, we hypothesised that the neural representation of auditory duration would be segmented. Thus, when it comes to the controversial role of alpha oscillation in WM, we inferred that if the alpha oscillation reflects different internal representation of auditory durations below and above the critical threshold point, then the alpha band reflects the successful maintenance of item information^3, 17, 23^. If there is no significant alpha band difference between durations below and above the critical threshold point, then the alpha band reflects inhibition of no-longer-relevant information^18, 21, 22^.

## Results

### Behavioural data

A repeated measures analysis of variance (ANOVA) was performed separately on accuracy and reaction time, with duration (1 s, 2 s, 3 s, and 4 s) as a within-participant factor. There was a significant main effect of duration [*F*(2.071,49.698) = 61.205, *p* < 0.001, *η*_*p*_^2^ = 0.718] on accuracy. Results of post hoc tests showed that the accuracy of the 1-s condition (mean ± standard error: 0.888 ± 0.017) was significantly higher than that of the 2-s (0.840 ± 0.019), 3-s (0.729 ± 0.023), and 4-s (0.670 ± 0.028) conditions (*t*(24) = 3.961–9.216, *p* values < 0.01); the accuracy of the 2-s condition was significantly higher than that of the 3-s and 4-s conditions (*t*(24) = 7.232–8.881, *p* values < 0.001); and the difference between the 3-s and 4-s conditions was also significant (*t*(24) = 3.596, *p* < 0.01).

There was also a significant effect of duration on reaction time [*F*(3,72) = 3.176, *p* < 0.05, *η*_*p*_^2^ = 0.117]. Results of the post hoc tests demonstrated that the difference between the 1-s (449.885 ± 22.788) and 2-s (457.184 ± 25.217) conditions on reaction time was not significant (*t*(24) = −1.090, *p* > 0.05), whereas there was a marginal significant difference between the 1-s and 3-s (467.358 ± 27.116) conditions (*t*(24) = −2.042, *p* < 0.06). In addition, the reaction time of the 1-s condition was significantly shorter than that of the 4-s (469.806 ± 27.463) condition. Differences among the 2-s, 3-s, and 4-s conditions were not significant (*t*(24) = −1.756–0.413, *p* values > 0.05).

### EEG data

**Figure 2:**
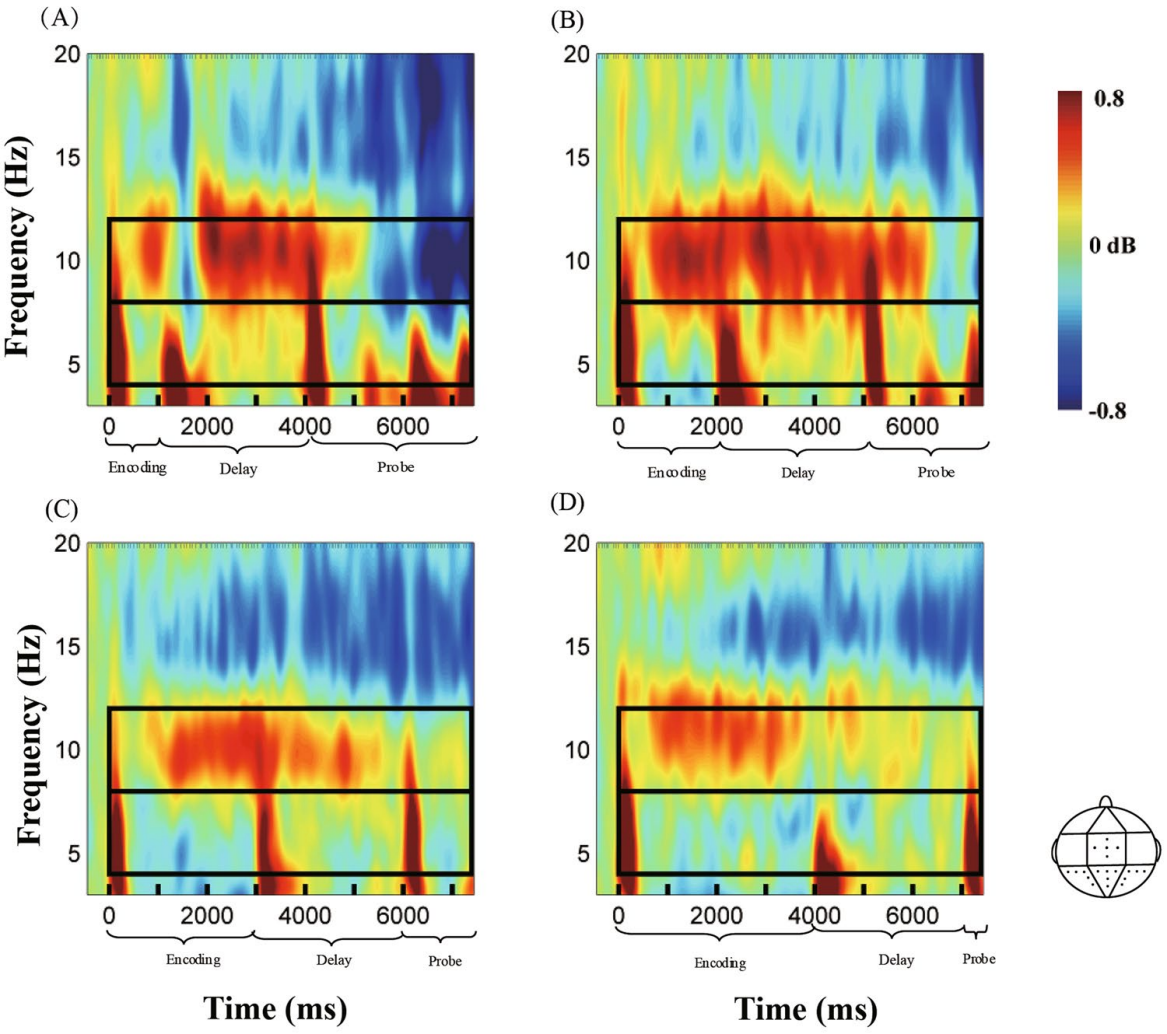
Theta and alpha effects for the whole epoch in the posterior parietal in the 1-s (**A**), 2-s (**B**), 3-s (**C**), and 4-s (**D**) auditory duration conditions. The decibel-transformed value is relative to the baseline interval (−0.4 s to −0.1 s) before the sample stimulus. The theta band is enhanced at the onset and offset of stimuli, whereas the alpha band is enhanced during encoding, especially in the delay phase.

**Figure 3:**
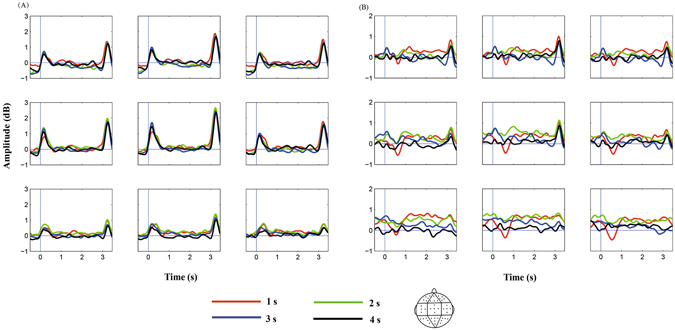
Average theta (**A**) and alpha (**B**) band powers during the 1-s, 2-s, 3-s, and 4-s auditory duration maintenance in WM. Steady theta (4–8 Hz) and alpha (8–12 Hz) band activities are elicited during WM maintenance after averaging across frequency, and they are separately plotted for each of the nine analysed electrode clusters. Zero on the x-axis represents the onset of the delay phase. Red, green, blue, and black curves denote the 1-s, 2-s, 3-s, and 4-s auditory duration conditions, respectively.

**Figure 4:**
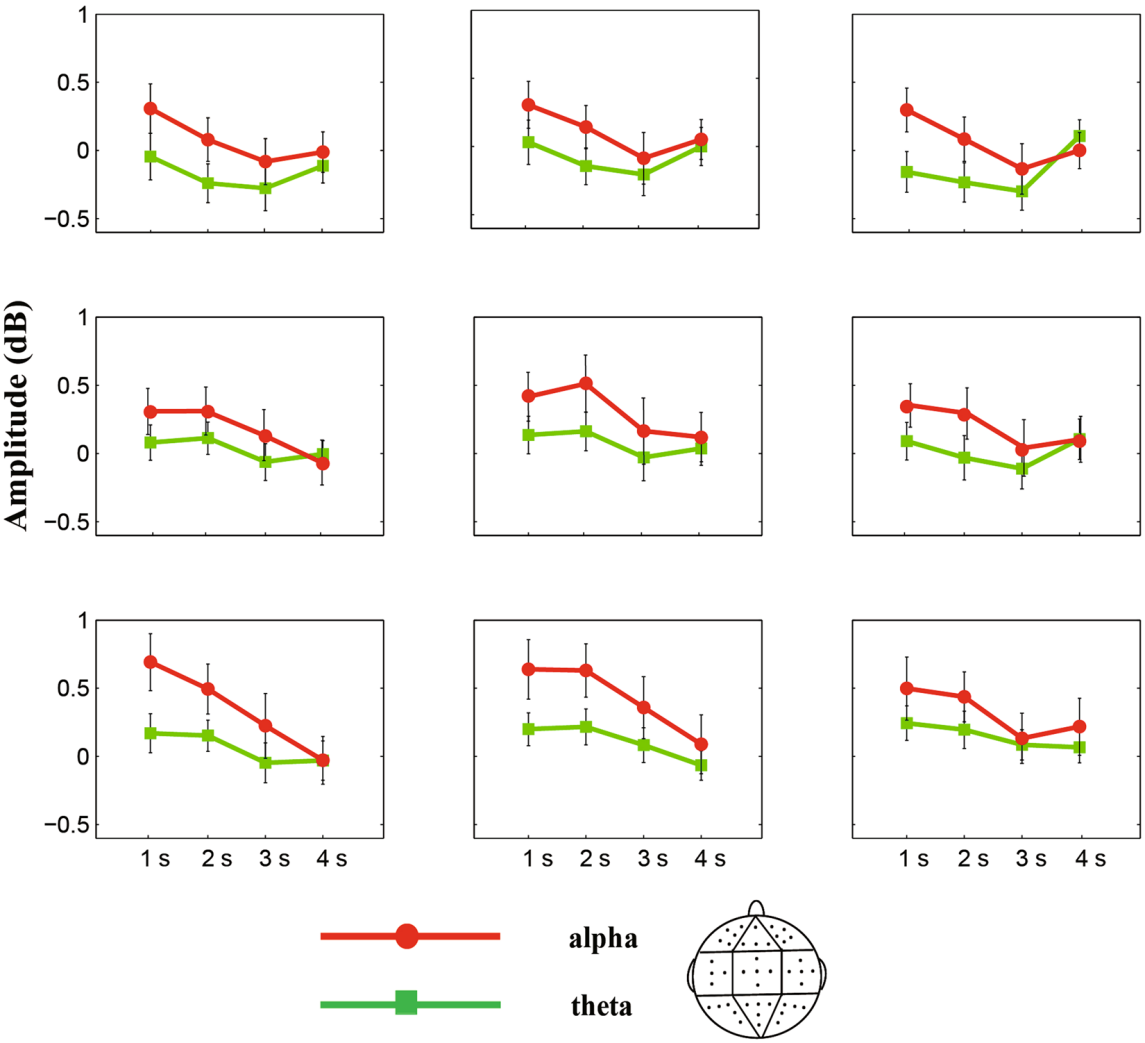
Grand average theta and alpha band powers during auditory duration maintenance in WM in the 1-s, 2-s, 3-s, and 4-s conditions. Grand average theta and alpha band powers during the delay phase are computed for each duration condition, and they are separately plotted for each of the nine analysed electrode clusters. Red and green lines denote the alpha and theta band powers in each duration condition, respectively. Error bars represent the standard error of the mean across observers.

Figure 2 shows the dynamic activities of theta and alpha powers during the encoding (sample), delay, and probe phases. Figure 3 shows the average theta and alpha band powers during the time course of the delay phase. Repeated-measures ANOVA on the theta band power from the interval of 0.5 s to 0.1 s before the onset of delay found no significant effect of duration or a duration × region interaction (*p* values > 0.05). An ANOVA conducted on the theta band power from the 1-s to 3-s interval after the onset of delay found a significant main effect of region [*F*(2.207,52.959) = 3.524, *p* < 0.05, *η*_*p*_^2^ = 0.128]. The regions with the highest theta band power during WM maintenance were the right-posterior cluster (0.148 ± 0.098 dB), middle-posterior cluster (0.108 ± 0.100 dB), middle-central cluster (0.076 ± 0.119 dB), and left-posterior cluster (0.061 ± 0.109 dB). There was no significant effect of duration [*F*(3,72) = 1.321, *p* > 0.05, *η*_*p*_^2^ = 0.052] or a duration × region interaction [*F*(8.316,199.582) = 1.475, *p* > 0.05, *η*_*p*_^2^ = 0.058] (Fig. 4).

Repeated-measures ANOVA conducted on the alpha band power from the interval of 0.5 s to 0.1 s before the onset of delay demonstrated no significant effect of duration or a duration × region interaction (*p* values > 0.05). An ANOVA conducted on the alpha band power from the interval of 1 s to 3 s after the onset of delay found a significant main effect of duration [*F*(3,72) = 3.011, *p* < 0.05, *η*_*p*_^2^ = 0.111]. Results of post hoc tests showed that the difference of the alpha power between the 1-s (0.426 ± 0.170 dB) and 2-s (0.333 ± 0.153 dB) conditions was not significant (*t*(24) = 0.643, *p* > 0.05). Moreover, the alpha power for these duration conditions were higher (or marginal higher) than that of the 3-s (0.084 ± 0.185 dB) and 4-s (0.053 ± 0.153 dB) conditions (*t*(24) = 1.899–2.459, *p* values < 0.07), and the difference between the 3-s and 4-s conditions was not significant (*t*(24) = 0.203, *p* > 0.05). The main effect of the region was significant [*F*(3.751,90.031) = 3.807, *p* < 0.01, *η*_*p*_^2^ = 0.137], and the regions with the highest alpha band power during WM maintenance were the middle-posterior cluster (0.429 ± 0.183 dB), left-posterior cluster (0.346 ± 0.157 dB), right-posterior cluster (0.321 ± 0.170 dB), and middle-central cluster (0.304 ± 0.175 dB). The duration × region interaction [*F*(8.655,207.714) = 1.521, *p* > 0.05, *η*_*p*_^2^ = 0.060] was not significant (Fig. 4).

**Figure 5:**
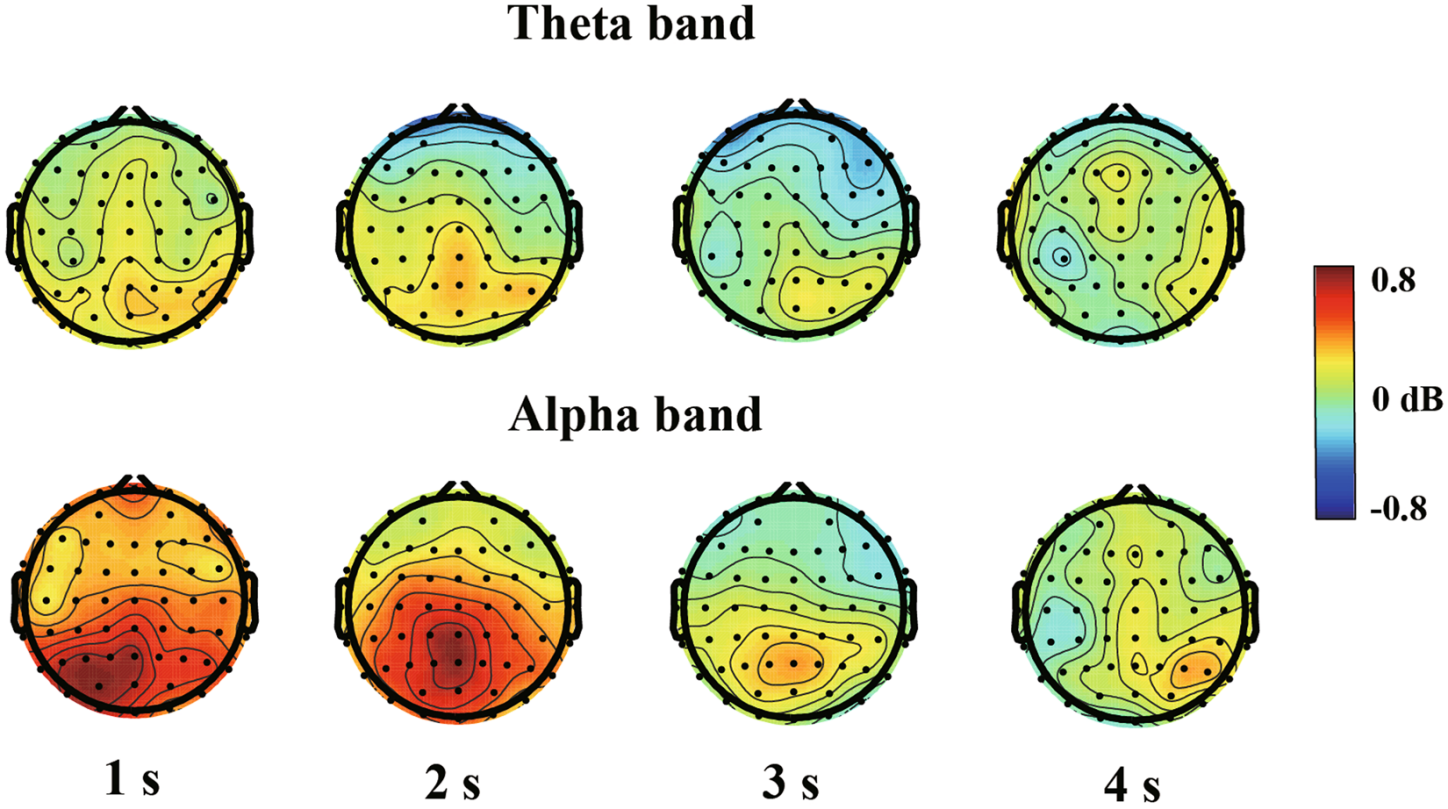
Topographies of theta and alpha activity during auditory duration maintenance in working memory in the 1-s, 2-s, 3-s, and 4-s conditions. Theta and alpha band powers are significantly activated in the posterior parietal cortex from the interval of 1 s to 3 s after the onset of delay.

**Figure 6:**
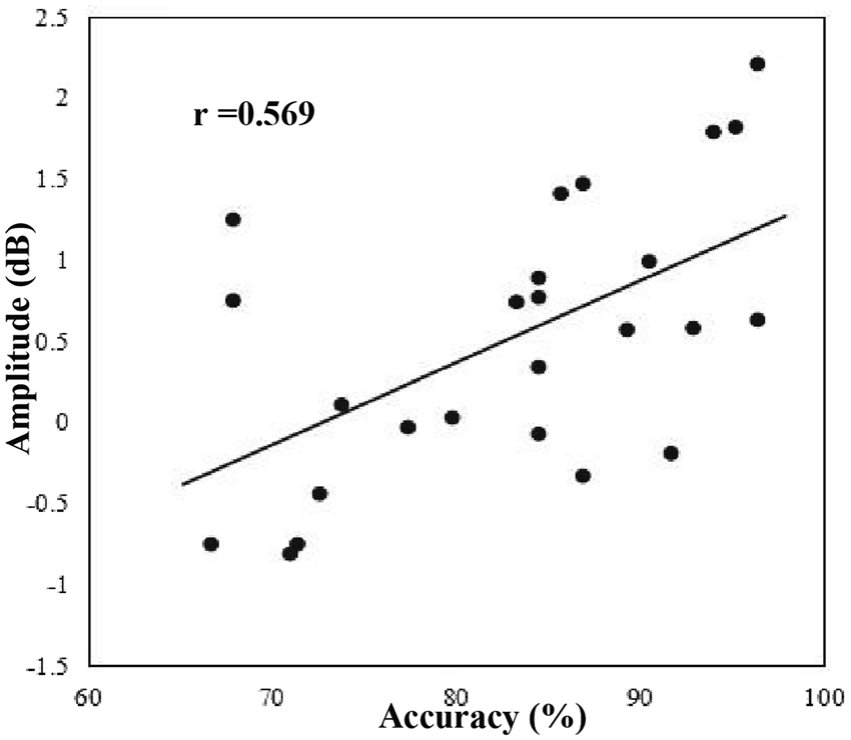
Correlation between the amplitude of the alpha band power and accuracy in the posterior parietal during the 2-s auditory duration condition. The scatter plot depicts the amplitude of the alpha power (y*-*axis) relative to the accuracy of the behavioural performance (x-axis) in the 2-s auditory duration condition. Pearson product-moment correlation coefficient, *r* = 0.569.

Figure 5 shows that the region with the highest theta and alpha band powers during WM maintenance were the posterior parietal (i.e. the left-posterior cluster, middle-posterior cluster, right-posterior cluster, and middle-central cluster). A correlation analysis was performed to determine the relationship between the amplitude of the alpha power and the accuracy at the posterior parietal cortex (i.e. 20 channels in total) during the delay phase for every duration condition to further examine the role of the alpha band power in auditory duration maintenance in WM. As shown in Fig. 6, we found that the amplitude of the alpha power was positively correlated with the accuracy of the 2-s condition (Pearson *r* = 0.569, *p* < 0.01). In contrast, the correlations for the 1-s (*r* = 0.155, *p* > 0.05), 3-s (*r* = 0.094, *p* > 0.05) and 4-s conditions (*r* = −0.027, *p* > 0.05) were not significant.

## Discussion

Using a matching-to-sample task, the present study was to investigate the neural oscillations associated with auditory duration maintenance in WM. EEG analyses indicated that the auditory duration length was not associated with changes in the theta band amplitude, which confirmed our hypothesis that the theta band is not involved in auditory duration maintenance in WM. The alpha band amplitudes during 3-s and 4-s auditory duration conditions were lower than during the 1-s and 2-s conditions. Additionally, the alpha amplitude positively correlated with accuracy in the 2-s condition. These results emphasise the involvement of the alpha band in auditory duration maintenance in WM. They are also consistent with our hypothesis that the neural representation of auditory duration is segmented with a critical point of approximately 2 s, which is shorter than that for visual duration (3 s).

The neural representations of different lengths of durations are distinct. Separate mechanisms process durations below and above the critical threshold point. Compared with the results of Chen *et al*.’s^3^ study on visual duration, our study indicated that there are different threshold points between different sensory modalities. As mentioned earlier, only duration below the critical threshold point is represented as a coherent experience or temporal gestalt in WM^25^. However, auditory signals are often subjectively perceived to be longer than visual ones for a given duration^31–35^. As described in the pacemaker-accumulator model of timing^39^, a pacemaker in the brain emitted pulses at the onset of a timed auditory/visual stimulus, and these pulses were summed by an accumulator until the stimulus stopped. Perception of duration depends on the number of pulses counted in the accumulator. Some researchers claim that the rate of internal clock for auditory signals is faster than that for visual ones^33, 34^. Others hold that it is better for auditory signals to capture attention than visual ones^32, 40^. In both cases, the accumulated clock value of auditory signals will be larger; therefore, the critical point of auditory duration in WM will be shorter compared with visual duration.

The role of the alpha oscillation is controversial, and our study’s findings supported that the alpha band is related to successful maintenance of item information. The most important reason is that the ‘inhibition’ hypothesis of the alpha band cannot explain the separate representations of auditory duration below and above 2 s. Every duration condition should be inhibited in the same way if alpha band reflects inhibition of no-longer-relevant information^18, 21, 22^, and there should be no significant alpha band difference between durations below and above the critical threshold point. Furthermore, our results indicated that the alpha band power was greatest over the posterior parietal during WM maintenance, which was suggested to be important to the storage of information in WM^41, 42^. One EEG study also reported that alpha band activities distributed over the posterior parietal and lateral occipital were increased during item maintenance in WM, and the enhancement was primarily evident in high performers on the item WM task^23^. The parietal cortex is also considered as the most important region associated with temporal processing^43^. In addition, individual threshold differences are likely to exist even though the general threshold is 2 s. That is, individuals with a longer threshold (2 s is a short duration) likely show a high accuracy and a high alpha band amplitude if the alpha band is engaged in the maintenance of auditory duration in WM. In contrast, individuals with a shorter threshold (2 s is a long duration) probably show a low discrimination accuracy and a low alpha band amplitude. Indeed, we found a positive relationship between the alpha amplitude and accuracy in the 2-s auditory duration condition (Fig. 6). Previous studies have considered that WM-related alpha oscillations during WM maintenance are an essential constituent that sustains the neural representations of memorised items^44^, and they may preserve the fidelity of stimulus representations during WM maintenance^23^. It seems that the alpha oscillation fluctuations during stimulus maintenance represent complex network activity of internally directed attention that promote WM processes to enhance the stimulus maintenance^45^.

Four issues must be noted. First, there may be varying degrees of task difficulty among 1-s, 2-s, 3-s, and 4-s duration conditions. For example, determining the difference of a 1-s sample and 2-s probe could be more difficult than that of a 1-s sample and 4-s probe. Moreover, the responses were chosen from two options (i.e. equal or longer/shorter) for 1-s and 4-s samples and three options (i.e. equal, longer, or shorter) for 2-s and 3-s samples. Thus, it would probably have been easier to make a decision for 1-s and 4-s samples than 2-s and 3-s. However, the behavioural result showed that the accuracy of judging significantly decreased with the increase of sample durations. This shows that the main conclusion was not affected by the task difficulty. Second, the time intervals between the baseline and delay phase among the 1-s, 2-s, 3-s, and 4-s conditions were different, which suggests the influence of the alpha amplitude in the delay phase. Thus, the alpha amplitude between −0.5 and −0.1 s before the onset of the delay phase may have been affected by another factor, namely the time intervals. However, an ANOVA found no significant main effect of duration or a duration × region interaction for the theta and alpha band amplitude during the interval between −0.5 and −0.1 s before the onset of the delay phase. This excluded the likelihood of an effect of the different time intervals between the baseline and delay phase on the theta and alpha band amplitude during the delay phase. Thus, the behavioural results did not support the presence of a threshold point between short and long auditory durations, which was different from the EEG results. This observation is likely due to the limitations of behavioural studies. Previous studies have suggested that variability in timing behaviour is mainly attributed to encoding, memory, and decision-making processes^39, 46^. Thus, WM as well as encoding and decision-making processes influenced the behavioural results of our study. EEG technology, nevertheless, can distinguish processes of encoding, maintenance, and decision-making for temporal information. Clearly, EEG data are more suitable than behavioural data for investigating a threshold point between short and long durations^3^. Finally, there are some other differences between the neural representation of visual and auditory durations in addition to the difference of the critical threshold point (e.g. the encoding-related alpha oscillations and the topography of WM maintenance). This probably indicates that WM for stimulus duration is organised by stimulus modality, as predicted in previous behavioural studies^7, 9, 10^. On the basis of the advantages of electroencephalography technology, the present study has partly revealed the differences between visual and auditory durations in the neural representation compared to Chen *et al*.’s study^3^. However, this is still a preliminary assessment. Further detailed studies of various advantageous techniques (e.g. functional magnetic resonance imaging with high spatial resolution) are still needed.

## Methods

### Participants

Twenty-five right-handed undergraduates (9 men, 21.00 ± 1.78 years old) were paid for their participation in the present study. Each participant provided written informed consent, and they had normal or corrected-to-normal vision. Our study received approval from the local institutional review board of Southwest University and was compliant with the ethical standards of the Declaration of Helsinki^47^.

### Stimuli and apparatus

Auditory stimuli were 1, 2, 3, or 4-s pure tones of 1,000 Hz that were binaurally presented through Sennheiser stereo earphones. The loudness was adjusted to be comfortable to the subjects at ~60 dB. The response signal was a 2-cm white question mark. The computer screen was positioned approximately 75 cm from the participants’ eyes. The refresh rate of the monitor was 85 Hz.

### Procedures

We used a classical matching-to-sample task (Fig. 1). Participants first heard a pure tone (the sample stimulus), followed by a 3-s interval (the delay/maintenance phase). Next, a second pure tone (the probe stimulus) was presented. Each tone was randomly presented for 1, 2, 3, or 4 s. After a 1-s interval, a question mark (response signal) was displayed on the screen until a key was pressed, or for a maximum of 2 s. During the response period, participants were instructed to press ‘1’, ‘2’, or ‘3’ correspondingly after estimating the duration of the second pure tone (probe) as shorter, equal to, or longer than the first tone (sample). Half of the participants responded with their left hand, and the other half responded with their right hand. The intertrial interval was 2 s. There were seven blocks with 48 trials in each block. Every duration condition contained 84 trials.

### Electrophysiological recording

A continuous EEG was acquired from a 64-channel scalp cap (Brain Products GmbH, Herrsching, Germany) at a rate of 500 Hz, which conformed to the extended 10–20 system of channel locations with an amplifier bandpass of 0.01–100 Hz, including a 50-Hz notch filter. Additional electrodes were positioned on the left and right mastoids. The horizontal and vertical electrooculogram were acquired using electrodes positioned at the external ocular canthi and below the right eye, respectively. Impedances were maintained below 5 kΩ for all electrodes.

### EEG analysis

All data processing was performed offline using EEGLAB^48^ and MATLAB (MathWorks, Natick, MA, USA). Continuous EEG data were re-referenced to the average of bilateral mastoids, and high-pass filtered at 0.5 Hz^3, 23, 38^. EEG epochs were extracted in 9-s time windows (pre-stimulus 1 s and post-stimulus 8 s, and zero was the onset of the sample stimulus). Each EEG epoch was baseline-corrected by subtracting the mean voltage before the sample stimulus. Epochs with an amplitude exceeding ± 100 μV were automatically marked and then manually confirmed and excluded through visual inspection. An independent component analysis was implemented to remove artefacts such as eye blinks and movements in terms of the scalp maps and activity profile^49, 50^. A total of 93.40% of the trials remained, and there was no significant main effect of duration (four duration conditions) on the remaining number of trials after ANOVA [*F*(2.144,51.455) = 0.103, *p* > 0.05, *η*_*p*_^2^ = 0.004].

A time-frequency analysis was performed for the segmented and artefact-free data, which used Hanning-windowed sinusoidal wavelets of three cycles at 3 Hz, increasing linearly to approximately 15 cycles at 30 Hz; a similar approach was used by Makeig *et al*.^51^ and Chen *et al*.^3^. It was used to optimise the trade-off between temporal resolution at lower frequencies and stability at higher frequencies by selecting the modified wavelet transform^51^. The event-related spectral perturbation (ERSP) index was adopted to compute the changes in event-related spectral power response (in dB)^52^, which was decibel-transformed relative to the baseline interval (−0.4 s to −0.1 s) before the sample stimulus. Figure 2 shows the ERSP during the encoding, delay, and probe phases of the sample duration relative to the baseline. The spectral analysis was based on single trials.

As shown in Fig. 3, steady theta (4–8 Hz) and alpha band (8–12 Hz) activities were elicited during WM maintenance after averaging across frequency. For this new coordinate, zero represents the onset of the delay phase. Another average was computed for the theta and alpha amplitude during the delay phase, which is shown in Fig. 4. Topographic EEG power analyses were implemented by grouping electrodes into nine clusters based on previous studies^38^, i.e. the left-frontal cluster (Fp1, AF3, AF7, F3, and F5), middle-frontal cluster (Fpz, F1, Fz, and F2), right-frontal cluster (Fp2, AF4, AF8, F4, and F6), left-central cluster (FC5, C3, C5, T7, and CP5), middle-central cluster (FCz, Cz, C1, C2, and CPz), right-central cluster (FC6, C4, C6, T8, and CP6), left-posterior cluster (P3, P5, P7, PO3, and O1), middle-posterior cluster (P1, P2, Pz, POz, and Oz), and right-posterior cluster (P4, P6, P8, PO4, and O2).

The time intervals were different between the baseline and delay phase among the four duration conditions. Therefore, we chose an additional statistical time window of 0.5 s to 0.1 s before the onset of delay to examine whether the following statistical analysis was contaminated by the different time intervals. A two-way repeated measures ANOVA was successively performed on the mean power of the theta and alpha bands during 0.5 s to 0.1 s and 1 s to 3 s. Duration (1 s, 2 s, 3 s, and 4 s) and region (nine clusters) were the ANOVA factors. We adopted the Greenhouse-Geisser correction method to correct for any violations of sphericity^53^, and we used partial eta squared (*η*_*p*_^2^) to estimate the ANOVA effect size^54^.

## References

[1] Saint Augustine (Bishop of Hippo.). The Confessions of Saint Augustine (ed. T. Scott) 123 (Modern Library, 1949).

[2] Baddeley A (1992). Working memory. Science.

[3] Chen Y, Chen X, Kuang C, Huang X (2015). Neural oscillatory correlates of duration maintenance in working memory. Neuroscience.

[4] Penney, T. B. Modality differences in interval timing: Attention, clock speed, and memory (ed. W. H. Meck) 209–234 (CRC Press, 2003).

[5] Plummer C, Humphrey N (2009). Time perception in children with ADHD: The effects of task modality and duration. Child Neuropsychology.

[6] Rammsayer TH, Borter N, Troche SJ (2015). Visual-auditory differences in duration discrimination of intervals in the subsecond and second range. Frontiers in psychology.

[7] Rattat AC, Picard D (2012). Short-term memory for auditory and visual durations: Evidence for selective interference effects. Psychological research.

[8] Wearden J, Parry A, Stamp L (2002). Is subjective shortening in human memory unique to time representations?. The Quarterly Journal of Experimental Psychology: Section B.

[9] Franssen V, Vandierendonck A, Van Hiel A (2006). Duration estimation and the phonological loop: Articulatory suppression and irrelevant sounds. Psychological research.

[10] Rattat AC (2010). Bidirectional interference between timing and concurrent memory processing in children. Journal of Experimental Child Psychology.

[11] Jensen O (2006). Maintenance of multiple working memory items by temporal segmentation. Neuroscience.

[12] Koene RA, Hasselmo ME (2007). First-In–First-Out Item Replacement in a Model of Short-Term Memory Based on Persistent Spiking. Cerebral Cortex.

[13] Lisman JE, Idiart MA (1995). Storage of 7 plus/minus 2 short-term memories in oscillatory subcycles. Science.

[14] Chen Y, Huang X (2016). Modulation of Alpha and Beta Oscillations during an n-back Task with Varying Temporal Memory Load. Frontiers in psychology.

[15] Gevins A, Smith ME, McEvoy L, Yu D (1997). High-resolution EEG mapping of cortical activation related to working memory: effects of task difficulty, type of processing, and practice. Cerebral Cortex.

[16] Jensen O, Tesche CD (2002). Frontal theta activity in humans increases with memory load in a working memory task. European journal of Neuroscience.

[17] Johnson JS, Sutterer DW, Acheson DJ, Lewis-Peacock JA, Postle BR (2011). Increased alpha-band power during the retention of shapes and shape-location associations in visual short-term memory. Frontiers in psychology.

[18] Jokisch D, Jensen O (2007). Modulation of gamma and alpha activity during a working memory task engaging the dorsal or ventral stream. The Journal of Neuroscience.

[19] Meltzer JA, Negishi M, Mayes LC, Constable RT (2007). Individual differences in EEG theta and alpha dynamics during working memory correlate with fMRI responses across subjects. Clinical Neurophysiology.

[20] Tuladhar AM (2007). Parieto‐occipital sources account for the increase in alpha activity with working memory load. Human brain mapping.

[21] Klimesch W, Sauseng P, Hanslmayr S (2007). EEG alpha oscillations: the inhibition–timing hypothesis. Brain research reviews.

[22] Manza P, Hau CLV, Leung HC (2014). Alpha power gates relevant information during working memory updating. The Journal of Neuroscience.

[23] Hsieh LT, Ekstrom AD, Ranganath C (2011). Neural oscillations associated with item and temporal order maintenance in working memory. The Journal of Neuroscience.

[24] Fraisse P (1984). Perception and estimation of time. Annual review of psychology.

[25] Pöppel E (1997). A hierarchical model of temporal perception. Trends in cognitive sciences.

[26] Elbert T, Ulrich R, Rockstroh B, Lutzenberger W (1991). The Processing of Temporal Intervals Reflected by CNV‐Like Brain Potentials. Psychophysiology.

[27] Morillon B, Christian AK, Giraud A-L (2009). Three stages and four neural systems in time estimation. Journal of Neuroscience.

[28] Szelag E, von Steinbüchel N, Reiser M, Gilles DLE, Pöppel E (1995). Temporal constraints in processing of nonverbal rhythmic patterns. Acta Neurobiologiae Experimentalis.

[29] Wittmann M, Pöppl E (2000). Temporal mechanisms of the brain as fundamentals of communication—with special reference to music perception and performance. Musicae Scientiae.

[30] Mates J, Müller U, Radil T, Pöppel E (1994). Temporal integration in sensorimotor synchronization. Journal of cognitive neuroscience.

[31] Goldstone S, Lhamon WT (1974). Studies of auditory-visual differences in human time judgment: I. Sounds are judged longer than lights. Perceptual and motor skills.

[32] Penney TB, Gibbon J, Meck WH (2000). Differential effects of auditory and visual signals on clock speed and temporal memory. Journal of Experimental Psychology: Human Perception and Performance.

[33] Wearden JH, Edwards H, Fakhri M, Percival A (1998). Why“sounds are judged longer than lights”: Application of a model of the internal clock in humans. The Quarterly Journal of Experimental Psychology: Section B.

[34] Wearden J, Todd N, Jones L (2006). When do auditory/visual differences in duration judgements occur?. The Quarterly Journal of Experimental Psychology.

[35] Droit-Volet S, Meck WH, Penney TB (2007). Sensory modality and time perception in children and adults. Behavioural Processes.

[36] Coull JT, Nazarian B, Vidal F (2008). Timing, storage, and comparison of stimulus duration engage discrete anatomical components of a perceptual timing network. Journal of cognitive neuroscience.

[37] Harrington DL, Zimbelman JL, Hinton SC, Rao SM (2010). Neural modulation of temporal encoding, maintenance, and decision processes. Cerebral Cortex.

[38] Roberts BM, Hsieh L-T, Ranganath C (2013). Oscillatory activity during maintenance of spatial and temporal information in working memory. Neuropsychologia.

[39] Gibbon J, Church R. M. Sources of variance in an information processing theory of timing. In: Animal cognition (ed. Roitblat H. L., *et al*.). 465–488 (Erlbaum, 1984).

[40] Meck WH (1984). Attentional Bias between Modalities: Effect on the Internal Clock, Memory, and Decision Stages Used in Animal Time Discriminationa. Annals of the New York Academy of sciences.

[41] Hamidi M, Tononi G, Postle BR (2008). Evaluating frontal and parietal contributions to spatial working memory with repetitive transcranial magnetic stimulation. Brain research.

[42] Luber B (2007). Facilitation of performance in a working memory task with rTMS stimulation of the precuneus: frequency-and time-dependent effects. Brain research.

[43] Bueti D, Walsh V (2009). The parietal cortex and the representation of time, space, number and other magnitudes. Philosophical Transactions of the Royal Society of London B: Biological Sciences.

[44] Palva S, Palva JM (2007). New vistas for α-frequency band oscillations. Trends in neurosciences.

[45] Wilsch A, Obleser J (2016). What works in auditory working memory? A neural oscillations perspective. Brain research.

[46] Allan LG (1998). The influence of the scalar timing model on human timing research. Behavioural Processes.

[47] World Medical Association. WMA declaration of Helsinki–ethical principles for medical research involving human subjects. Available from: http://www.wma.net/en/30publications/10policies/b3/index.Html (2013). 10.1001/jama.2013.281053 24141714

[48] Delorme A, Makeig S (2004). EEGLAB: an open source toolbox for analysis of single-trial EEG dynamics including independent component analysis. Journal of neuroscience methods.

[49] Jung TP (2000). Removing electroencephalographic artefacts by blind source separation. Psychophysiology.

[50] Jung TP (2000). Removal of eye activity artefacts from visual event-related potentials in normal and clinical subjects. Clinical Neurophysiology.

[51] Makeig S (2004). Electroencephalographic brain dynamics following manually responded visual targets. PLoS Biol.

[52] Makeig S (1993). Auditory event-related dynamics of the EEG spectrum and effects of exposure to tones. Electroencephalography and clinical neurophysiology.

[53] Greenhouse SW, Geisser S (1959). On methods in the analysis of profile data. Psychometrika.

[54] Levine TR, Hullett CR (2002). Eta squared, partial eta squared, and misreporting of effect size in communication research. Human Communication Research.

